# Multi-Scale X-ray Imaging of the Pigment Discoloration Processes Triggered by Chlorine Compounds in the Upper Basilica of Saint Francis of Assisi

**DOI:** 10.3390/molecules28166106

**Published:** 2023-08-17

**Authors:** Ermanno Avranovich Clerici, Steven de Meyer, Frederik Vanmeert, Stijn Legrand, Letizia Monico, Costanza Miliani, Koen Janssens

**Affiliations:** 1 Antwerp X-ray Imaging and Spectroscopy Laboratory (AXIS) Research Group, NANOLab Centre of Excellence, University of Antwerp, Groenenborgerlaan 171, 2020 Antwerp, Belgium; steven.demeyer@uantwerpen.be (S.d.M.); frederik.vanmeert@uantwerpen.be (F.V.); stijn.legrand@uantwerpen.be (S.L.); letizia.monico@cnr.it (L.M.); koen.janssens@uantwerpen.be (K.J.); 2 Paintings Laboratory, Royal Institute for Cultural Heritage (KIK-IRPA), Jubelpark 1, 1000 Brussels, Belgium; 3 Scientific Methodologies Applied to Archaeology Centre of Excellence (SMAArt), Department of Chemistry, Biology and Biotechnology, University of Perugia, Via Elce di Sotto 8, 06123 Perugia, Italy; 4 CNR-SCITEC, c/o Department of Chemistry, Biology and Biotechnology, University of Perugia, Via Elce di Sotto 8, 06123 Perugia, Italy; 5 CNR-ISPC, Institute of Cultural Heritage Sciences, Via Card. G. Sanfelice 8, 80134 Naples, Italy; costanza.miliani@cnr.it; 6 Rijksmuseum, Conservation and Restoration, P.O. Box 74888, 1070 DN Amsterdam, The Netherlands

**Keywords:** X-rays, discoloration, Assisi, Giotto, imaging, synchrotron, chlorine

## Abstract

In this paper, the chromatic alteration of various types of paints, present on mural painting fragments derived from the vaults of The Upper Basilica of Saint Francis of Assisi in Italy (12th–13th century), is studied using synchrotron radiation. Six painted mural fragments, several square centimeters in size, were available for analysis, originating from the ceiling paintings attributed to Cimabue and Giotto; they correspond to originally white, blue/green, and brown/yellow/orange areas showing discoloration. As well as collecting macroscopic X-ray fluorescence and diffraction maps from the entire fragments in the laboratory and at the SOLEIL synchrotron, corresponding paint cross-sections were also analyzed using microscopic X-ray fluorescence and powder diffraction mapping at the PETRA-III synchrotron. Numerous secondary products were observed on the painted surfaces, such as (a) copper tri-hydroxychloride in green/blue areas; (b) corderoite and calomel in vermillion red/cinnabar-rich paints; (c) plattnerite and/or scrutinyite assumed to be oxidation products of (hydro)cerussite (2PbCO_3_·Pb(OH)_2_) in the white areas, and (d) the calcium oxalates whewellite and weddellite. An extensive presence of chlorinated metal salts points to the central role of chlorine-containing compounds during the degradation of the 800-year-old paint, leading to, among other things, the formation of the rare mineral cumengeite (21PbCl_2_·20Cu(OH)_2_·6H_2_O).

## 1. Introduction

Throughout the history of art, Old Master painters were already aware of which artists’ pigments were the most durable and the most suitable to be applied in various contexts; this experience has been noted down in treatises by, e.g., the philosopher Theophrastus (c. 370–287 B.C.), the architect Vitruvius (c. 80–15 B.C.), the naturalist Pliny the Elder (c. 23–79), and the painter Cennino Cennini (c. 1360–1427).

Even though the knowledge about the proper and improper application of pigments was described in reference works on painting techniques and orally transferred from teacher to pupil, it is nowadays possible to observe the use of pigments that, several decades or centuries ago, were applied in environments that are not optimal for their chemical stability. A remarkable example of such practices can be observed in polychrome mural paintings of the Upper Basilica of Saint Francis in Assisi (Italy).

**Figure 1:**
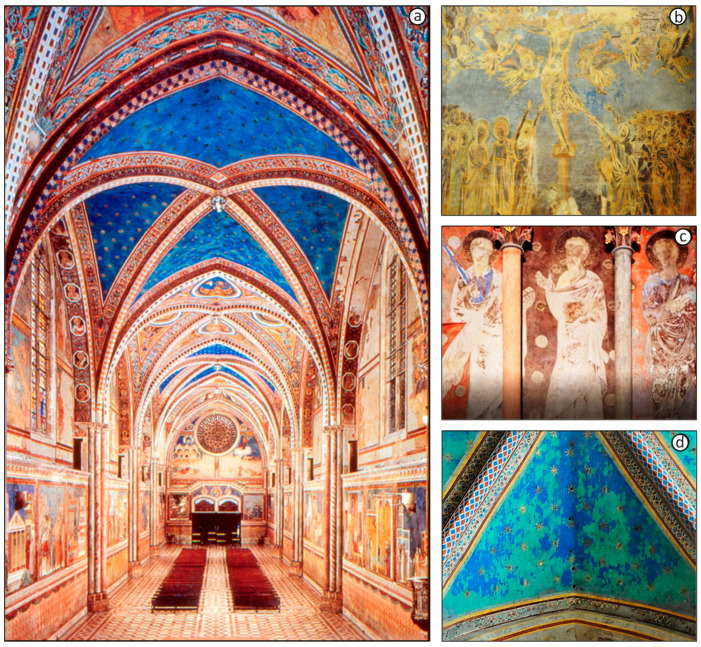
(**a**) View of the Upper Church in The Basilica of Saint Francis, Assisi, Italy. The intensely decorated walls and the Gothic style of the composition with its pointed arches can be observed. (**b**) “View of the Crucifixion”, a wall painting by Cimabue (c. 1283, Basilica of St. Francis, Assisi) in which a characteristic phenomenon of color inversion is visible, caused by the formation of black plattnerite from the original lead white. (**c**) Detail of the left loggia of the north transept, wall painting by the Northern Master or by Cimabue (late 13th century, Basilica of St. Francis, Assisi) depicting three apostles on a blackened red background, originally painted in vermilion. (**d**) View of the vault in which the star-studded sky, assumed to be painted by Giotto, shows random green patches affecting the original blue color (late 13th century, Basilica of St. Francis, Assisi).

More specifically, in this study, the discoloration of three important pigments: lead white (mainly constituted of hydrocerussite (2PbCO_3_·Pb(OH)_2_)—Figure 1b; vermilion red (HgS)—Figure 1c; and azurite (Cu_3_(CO_3_)_2_(OH)_2_)—Figure 1d was studied [1,2]. All these pigmented materials were applied on plaster to create wall paintings despite the fact it was known that these pigments should only be applied on canvas or on suitably prepared wooden panels. In what follows, we describe the results of studying the discoloration phenomena of these materials inside the Saint Francis Upper Basilica, allowing us to identify a Cl-containing oxidizer as the cause for the degradation that commonly affects all these three pigments.

Figure 1b–d illustrate various degradation processes that have occurred in some wall paintings of the Basilica. The blackening of lead white is a phenomenon that can be extensively observed in some of the masterpieces painted by Cimabue, such as The Crucifixion, in the Upper Church, in which a very striking color inversion is visible. Whereas originally the highlighted areas of the faces of the human figures and of their draped clothing were painted with lead white, these parts of the composition are now severely blackened. Thus, the originally shaded areas of the faces and drapery are now the lightest areas (see Figure 1b). Similarly, some of the red vermillion areas show a partial darkening to an unesthetic brownish color, as shown in Figure 1c. A very striking discoloration can be observed in the (originally blue) vault shown in Figure 1d, representing a star-studded sky, attributed to Giotto, where irregularly shaped patches have turned a pale green (Figure S2b).

**Figure 2:**
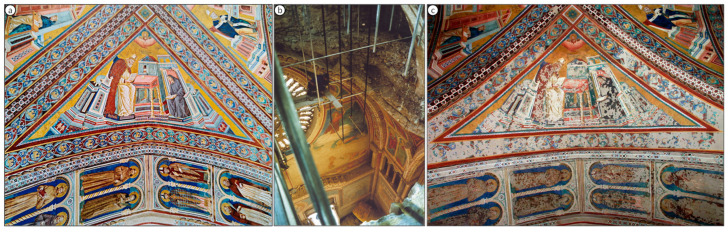
Photographs of the vaulted ceiling where Saint Jerome and the vaulting rib with the figures of eight saints were painted in the Upper Basilica of St. Francis in Assisi, Italy: (**a**) the outlook prior to the earthquakes in 1997, (**b**) view looking down through the ceiling of the nave after the earthquake (**c**) and after the restorations that ended in 2002.

**Figure 3:**
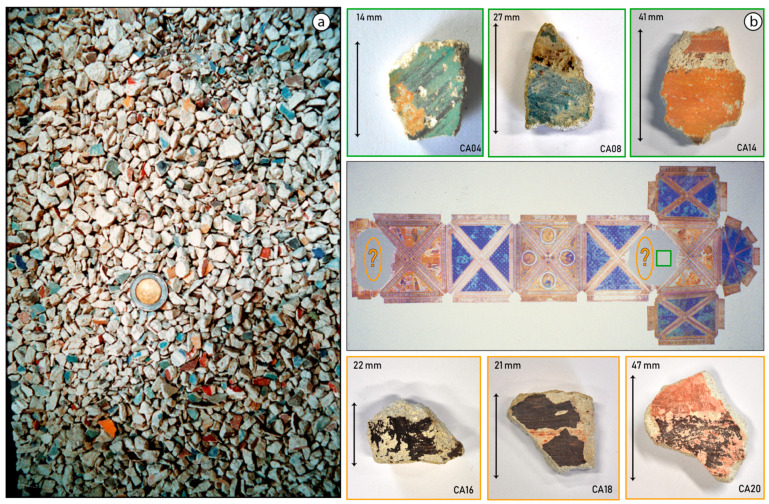
(**a**) Several thousand paint fragments a few cm^2^ in size were collected after the earthquake in 1997. A 2 Euro coin is included for scale. (**b**) On the right, a simplified scheme with a view of the vaults of the Upper Basilica of St. Francis in Assisi showing an indication (green square) of the origin of the top three painted mural painting fragments investigated. In the case of samples CA16, CA18, and CA20, as symbolically indicated by orange ellipses, the exact information about their original locations was not available. However, there is no doubt all samples in the lower row trace their origin to the collapsed vault paintings by Cimabue and/or his followers.

On 26 September 1997, two earthquakes hit the Assisi area in rapid succession. Most of the wall paintings of the Basilica were spared but a portion of the vaulting collapsed, as displayed in Figure 2b and Figure S3b. More than 300,000 painting fragments, shown in Figure 3a, were meticulously collected and individually cataloged before initiating an extensive selection and restoration process, as shown in Figure 2c, Figures S2a and S3c. This event provided the Central Institute for Restoration (ICR) in 2005–2006 with the first opportunity to examine the pigments employed in the mural paintings and to assess their state of alteration [2,3].

A subsequent investigation was performed by Vagnini et al. in 2018 [4], focusing on those fragments belonging to the scene dedicated to Saint Matthew.

In this study, the presence of a secondary product, formed during the darkening of lead white, was confirmed to be lead dioxide (plattnerite β-PbO_2_) even if, as underlined by the investigators, the artist used a *secco* painting technique instead of the *buon fresco* method of painting.

Another important observation made by Vagnini et al. is the ubiquitous presence of compounds containing at least one chlorine atom; for example, the transformation of azurite to basic copper chlorides (Cu_2_Cl(OH)_3_, i.e., the two polymorph forms atacamite and clinoatacamite) and the partial alteration of hematite (Fe_2_O_3_) to iron oxide chloride (FeOCl) were observed [4].

In order to investigate in greater detail the factors triggering the strong discolorations witnessed inside *The Upper Basilica of Saint Francis of Assisi* and identify possible common causes for these phenomena, in this work, several painted fragments were subjected to X-ray fluorescence and X-ray diffraction analyses. These measurements were performed to answer the following research questions:

1. Is there a similarity among the observed alteration phenomena? Can a single chemical agent be identified that is responsible for the discoloration of a variety of (white, red, and blue) pigments?
2. Is there a synergistic effect, i.e., are some degradation reactions caused/enhanced by a specific mixture of pigments applied together? Are there marker compounds for this?
3. Is it possible to identify degradation phenomena not only of the applied pigments but also of the binder and/or substrate? If so, do they show common patterns or are they influenced by surrounding compounds?

Bearing in mind the complexity of the palette used inside the Basilica and of the degradation processes expected to have taken place, it was considered highly relevant to record the distributions of primary and secondary compounds, with the goal to distinguish and document the relationships between the original pigments and of different secondary products that formed during the in-situ discoloration processes.

## 2. Materials and Methods

### 2.1. Mural Painting Fragments

Six painting fragments were investigated, shown in Figure 3b, with a surface area in the square centimeter range and in which an evident discoloration was visible. Three of the samples belonged to the Saint Matthew wall painting (marked with a green square in the ceiling scheme of the Upper Basilica, Figure 3b). The other three selected fragments had a less-clear provenance but undoubtedly formed part of the Upper Basilica’s ceiling until 1997 and were likely painted by Cimabue or his disciples (including Giotto). Following Vagnini et al. [4], all the pigments analyzed in this manuscript have been considered to be applied using the *secco* painting technique. In addition to the macroscopic analyses, small paint chips were collected from these centimeter-sized paint fragments and examined at the micro scale by employing chemical mapping methods based on synchrotron radiation to gain additional information regarding the build-up of the paint stratigraphy. In what follows, we will use the term *fragment* to refer to the entire (centimeter-sized) piece of painted plaster and the term *cross-section* to describe the polished and embedded micro-fragments of paint sampled from the main plaster fragments.

#### Paint Cross-Section Preparation

Paint micro-chips were taken from painted fragments of interest by means of a scalpel and embedded in *Epofix* resin (Struers, Westlake, Cleveland, OH, USA); the latter was left to dry for 24 h under vacuum. A rotary microtome (Microm HM 360 Automated Microtome, Marshall Scientific, Hampton, VA, USA) was used to cut sections from the resin blocks with a thickness between 30 and 50 µm.

### 2.2. Analytical Method

Two X-ray imaging techniques X-ray fluorescence (XRF) and X-ray powder diffraction (XRPD) were employed to analyze the samples with the aim of identifying and recording the distribution of the original pigment applied, to localize their by-products and determine if other crystalline compounds were present in adjacent areas.

The investigations explored different length scales to detail the circumstances necessary to promote the discoloration of intentionally applied pigments.

- Laboratory-based macroscopic (centimeter and millimeter range) element- and compound-specific distributions were obtained by analyzing the painted surface with MA-XRF and MA-XRPD setups, as in Figure S1a. MA-XRF analysis is a well-established technique for the non-invasive investigation of flat, painted surfaces in the cultural heritage sector [5]. The results obtained from this preliminary investigation, performed in the laboratory at the University of Antwerp, are shown in the Supplementary Information (Figures S3a–S9a).
- A second set of macroscopic elemental and phase maps from the mural painting fragments was collected at the PUMA beamline of the synchrotron SOLEIL (Saint-Aubin, France). Since the lateral resolution of this beamline is greatly superior to that of the laboratory-based MA-XRPD setup, distribution maps from smaller areas could be collected in greater detail (Figure S1b).
- From the macroscopic datasets collected, it was possible to identify sample subareas in which both the original pigments and their corresponding degradation products were present. Minute paint chips were then collected from these regions and embedded in resin to gain information on the in-depth distributions of primary and secondary products in the paint [6]. Paint stratigraphy analyses were performed by means of SR µ-XRF and SR µ-XRPD using the Hard X-ray Micro/Nano-Probe beamline P06 (PETRA III storage ring, DESY, Hamburg, Germany) from ad-hoc prepared cross-sections.

#### 2.2.1. Laboratory MA-XRF

The MA-XRF setup employed was constructed and optimized at the University of Antwerp. The XRF measurement head was mounted on a software-controlled motor stage and employed a rhodium anode (Rh-K_α_ 20.04 keV) transmission tube (Moxtek, Orem, UT, USA) operated at 50 kV and 1000 μA and a Vortex EX-90 SDD detector (Hitachi, Tokyo, Japan) positioned at a 50° angle with respect to the incident X-ray beam. A modified version of this instrument has been described by Alfeld et al. [7].

MA-XRF scans were collected to gain data on the paint surface in multiple positions following a matrix X–Y and plotting the data in distribution maps. The spatial distance between two data acquisitions was 300 µm and the collection time used for each spectrum was 300 ms. The spectral data obtained were processed through dynamic analysis, employing both the PyMCA [8] and Datamuncher [9] software packages, capable of creating distribution maps of chemical elements.

#### 2.2.2. Laboratory MA-XRPD

The MA-XRPD setup used in this investigation was an instrument built at the University of Antwerp [10]. The possibility to employ this laboratory instrument in reflection geometry made it possible to collect data from mural fragments with a thickness in the centimeter range. Similar to the MA-XRF instrument, the X-ray generator and the detector move in front of the paint surface thanks to a motorized stage. In this manner, it is possible to record crystal-phase distribution maps with a lateral resolution in the millimeter range (≈1.0 × 0.2 mm^2^). The X-ray mirror system attached to the X-ray tube provides a circular X-ray beam of ca. 150 µm radius. Nevertheless, in the reflection geometry, a shallow angle of incidence δ of the primary beam (of around 10 degrees) is employed, producing, in practice, an elongated beam footprint. Due to the beam shape, the acquisition is accomplished by scanning with a similar step size. The data collection was performed employing a monochromatic copper source IµS-HB (Incoatec, Geesthacht, Germany; Cu-K_α_: 8.04 keV). The X-Ray generation was operated at 50 kV and a current of 1000 µA. A planar imaging detector (Pilatus 200K, Dectris, Villigen, Switzerland) was employed to collect diffraction patterns. A triangulation telemeter was required to maintain the same distance between the X-ray source and the surface of the uneven fragments. The analyzed mural fragments typically have lateral dimensions in the centimeter range. The recorded phase information only pertains to the crystalline compounds present in the painted surface due to the shallow sampling depth in reflection geometry; it can be calculated using the following expression [11]:

$$d_{99\%}=\frac{ln(100)}{\mu_{m}\rho_{m}\left(\frac{1}{sin\;\left(2\theta -\delta \right)\;\;}+\frac{1}{sin\left(\delta \right)}\right)}$$

where *µ_m_* is the mass absorption coefficient of the irradiated material and *ρ_m_* its density. *δ* is the incident angle between the X-ray beam and the sample surface and 2*θ* is the scattering angle. For *δ* equal to 10°, *d*_99%_ varies from a few µm in strongly absorbing materials such as pure PbO_2_ to 50 µm for chemical elements with a lower mean atomic number containing materials such as calcite (CaCO_3_).

The diffraction data were recorded with a collection time of 10 s per acquisition point. The two-dimensional (2D) X-ray patterns collected at each acquisition point were azimuthally integrated into one-dimensional (1D) diffractograms employing the XRDUA software [12]. The crystalline phases in the 1D pattern were identified by comparing the data with a powder diffraction database, taking into account the observed diffraction peak positions (2*θ* angles) and their relative intensities. After integration, fitting was performed on the 1D diffractograms using a Rietveld model containing the XRD patterns of the recognized crystalline structures and ultimately plotted in the form of thermal color-scale maps, generating distribution maps that are specific for particular crystal phases.

#### 2.2.3. SR MA-XRF and MA-XRPD Mapping at PUMA Beamline, Synchrotron SOLEIL

The painted fragments were macroscopically (total area investigated in the mm^2^ range) analyzed at the PUMA beamline (Photons Utilisés pour les Matériaux Anciens) of the synchrotron SOLEIL (Paris, France) [13]. They were scanned with a monochromatic beam employing a primary photon energy of 10 keV, obtained by means of a Si (111) double-crystal monochromator (DCM). A Kirkpatrick Baez (KB) mirror system was employed to focus the beam, by achieving a size of 5 × 3 μm^2^ (h × v). In this geometry, similarly to the laboratory MA-XRPD setup, an elliptical-shaped area is probed because of the relatively shallow angle (8 degrees) of incidence of the primary beam, horizontally stretching the elliptical footprint of the beam to 50 × 3 μm^2^ (h × v). On the other hand, thanks to the PUMA photon flux of about 10^9^ ph/s, the scanning time per pixel could be reduced to 1 s in comparison to the 10 s dwell time required by the laboratory MA-XRPD system. No radiation damage was noticed during these measurements, consistent with previous observations on this subject [14]. All XRF and XRPD data were processed by employing the software packages PyMCA and Datamuncher [8,9]; PyFAI [15] was used to azimuthally integrate the XRD patterns and XRDUA [12] was employed to perform compound identification and execute quantitative analysis by Rietveld refinement. Because of the lack of a laser-distance meter to maintain a constant distance between the sample and the detector at the PUMA setup, the XRPD data were post-corrected by shifting the one-dimensional 2θ-spectra so that the XRD peaks of a number of known compounds (e.g., calcite, whewellite) appeared at their correct values.

#### 2.2.4. µ-XRF and µ-XRPD Mapping at the P06 Beamline at PETRA-III Synchrotron

Diffraction and fluorescence signals were collected from cross-sections at the microprobe hutch of the Hard X-ray Micro/Nano-Probe Beamline P06 of DESY (Hamburg, Germany). This beamline is dedicated to scanning X-ray microscopy with micro/nanoscopic spatial resolution [16]. A primary photon energy of 12.3 or 21 keV, which is selected by means of a Si (111) DCM, was employed for analyzing the paint cross-sections. The KB mirror system allowed us to focus the beam down to ca. 0.5 × 0.5 μm^2^ (h × v).

By means of a Vortex EM Si drift detector, on-the-fly scanning and acquisition of XRF data with millisecond dwell times per scan pixel was possible. A hybrid photon-counting imaging detector, the EIGER X 4M (Dectris Ltd., Baden, Switzerland), was positioned behind the sample for transmission XRPD measurements, allowing for the simultaneous acquisition of X-ray fluorescence (SR µ-XRF) and diffraction (SR µ-XRPD) data. Calibration of the diffraction setup was performed by means of LaB_6_ as a reference sample. Every sample was observed before and during the measurements by means of an optical microscope (Keyence, Itasca, IL, USA) equipped with a perforated mirror to allow the possibility to observe the sample under the same angle of the incident X-ray beam while keeping the entire setup still.

## 3. Results

### 3.1. General Observations

In Table 1, the chemical elements and the crystalline compounds identified by XRF and XRPD scans in each of the investigated fragments are summarized.

#### 3.1.1. Ground Layers

*Substrate.* Based on the XRF data (Table 1), in every fragment, calcium, potassium, and strontium were identified with a matching distribution in the lime plaster. Similarly, S-Kα signals were mainly identified in the plaster and in some cases within the painted surface; for example, in fragment CA16, the sulfur distribution is correlated with the distribution of iron, while in fragment CA18, it matches the distribution of mercury. The XRPD data reveal calcite (CaCO_3_) and gypsum (CaSO_4_·2H_2_O) as the main compounds occurring in the substrate of every sample.

Quartz (SiO_2_) was found to be present as dispersed grains in the lime plaster of each fragment, probably due to the sand that was often used as filler in the *intonaco*. In fragments CA14 and CA20, quartz was also noticed in the ochre-containing paint layers.

*Priming.* Iron and manganese were often recognized in the ground layer; sometimes, as in sample CA04, the presence of an iron-containing layer was highlighted by both macro and µ-XRF maps in direct contact with the one containing mainly calcium, and it was considered as a primer. XRPD measurements identified the presence of goethite (α-FeO(OH)) as the main crystalline compound in this layer, evidenced by XRF analysis.

In samples CA14 and CA20, the XRF spectra from these fragments show the presence of Sn correlated with the calcium-rich areas. The result suggests that tin was applied as a primer below the lead-containing pigment (Figure S9c,d). This hypothesis is also supported by the fact that tin is identified only in areas not shielded by the pigment on top, even though no corresponding crystalline compounds were recognized.

#### 3.1.2. Paint Layers

**Figure 4:**
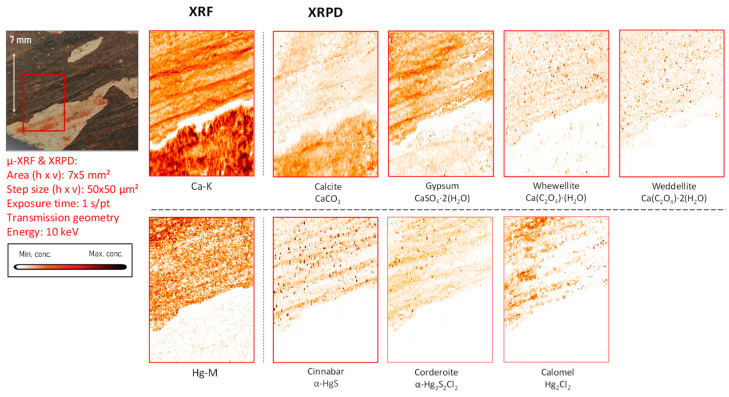
Dark-brown-colored vault fragment CA18,in red square the area analyzed and its corresponding MA-XRF maps of selected key elements, and MA-XRPD maps of selected primary and secondary products; data obtained at the PUMA beamline.

**Figure 5:**
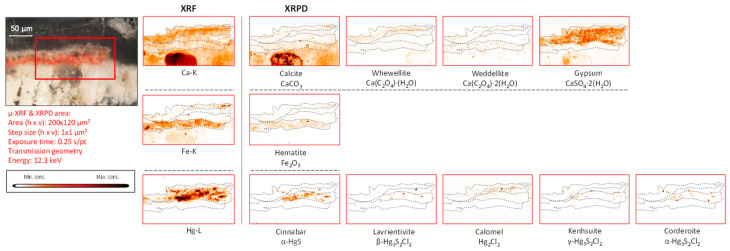
Cross-sectional micrograph of the paint stratigraphy in sample CA18 with its corresponding μ-XRF maps of selected elements and μ-XRPD maps of selected primary and secondary products; data obtained at Beamline P06.

*Mercury-based compounds:* The only sample in which XRF examinations revealed the occurrence of mercury is fragment CA18. Here, the entire pigmented area was found to be enriched in this element. From the X-ray powder diffraction data, it was possible to identify the mineral cinnabar as intentionally applied pigment together with its degradation products corderoite (Hg_3_S_2_Cl_2_) and calomel (Hg_2_Cl_2_) (Figure 4, Figure 5 and Figure S4).

**Figure 6:**
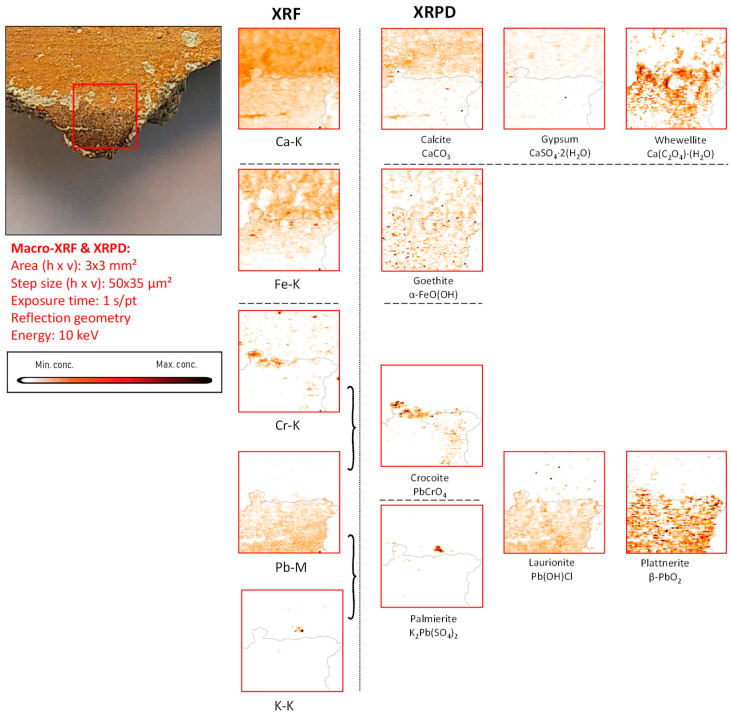
Orange-red colored vault fragment CA14, its corresponding MA-XRF maps of selected elements, and MA-XRPD maps of selected primary and secondary products; data obtained at the PUMA beamline.

**Figure 7:**
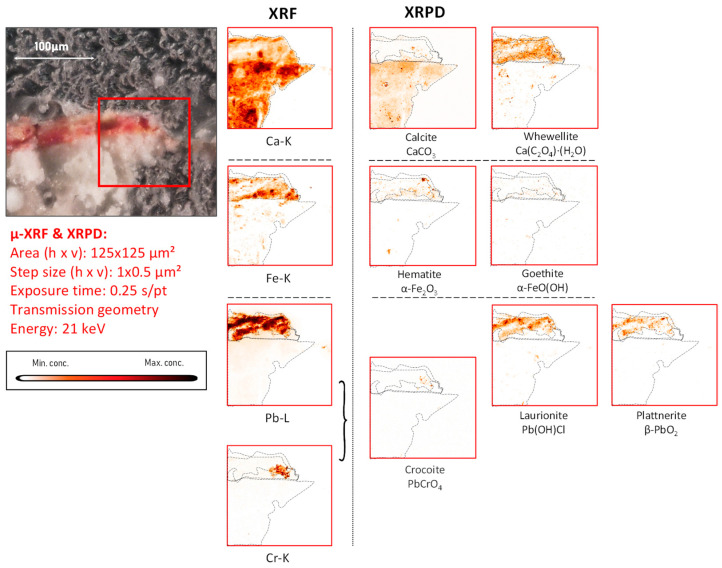
Cross-sectional micrograph of a paint sample collected from mural painting fragment CA14, its corresponding μ-XRF maps of selected elements, and μ-XRPD maps of selected primary and secondary products; data obtained at Beamline P06.

*Iron-based compounds.* In fragment CA14, the XRF data indicated an iron-containing earth pigment to be the main colorant visible to the naked eye, and XRPD data showed this to be a combination of hematite (α-Fe_2_O_3_) and goethite (α-FeO(OH)) employed, respectively, to a red and a yellow area. Titanium, identified in localized areas of the painted surfaces, is correlated to the presence of ochre, suggesting that it is an original part of this earth pigment (Figure 6, Figure 7 and Figure S5).

**Figure 8:**
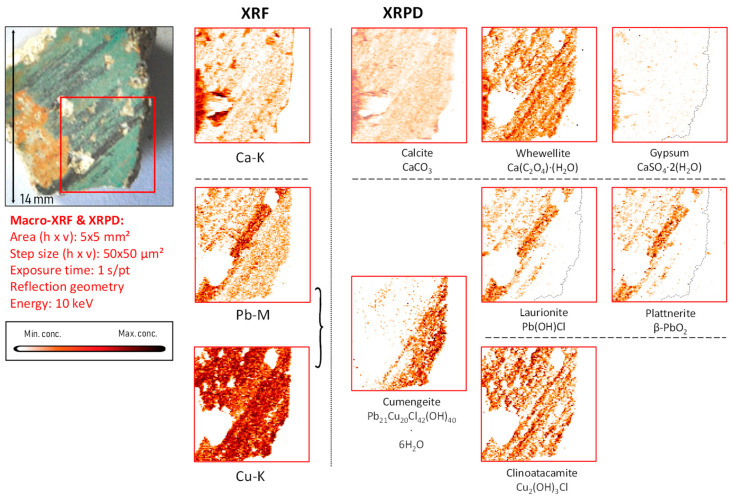
Green-black-colored vault fragment CA04, its corresponding MA-XRF maps of selected elements, and MA-XRPD maps of selected primary and secondary products; data obtained at the PUMA beamline.

**Figure 9:**
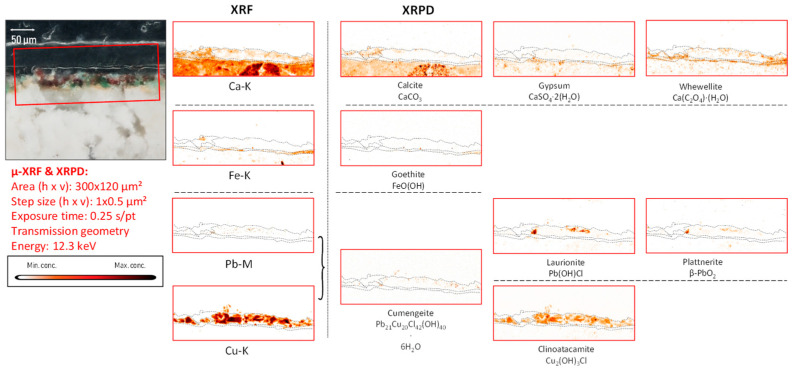
Cross-sectional micrograph of paint micro sample derived from mural painting fragment CA04, its corresponding μ-XRF maps of selected key elements, and μ-XRPD maps of selected primary and secondary products; data obtained at Beamline P06.

*Copper-based compounds.* In the copper-rich areas, chemical elements such as barium, zinc, arsenic, bismuth, and nickel were identified, occasionally dispersed in the entire paint stratigraphy or present in confined particles, as shown in Figure S8e. In these areas, the presence of copper tri-hydroxychlorides (Cu_2_(OH)_3_Cl) such as atacamite and clinoatacamite was detected (Figure 8, Figure 9, Figures S6 and S7). 

*Lead-based compounds.* Paint strokes containing lead were observed in three fragments (CA04, CA08, and CA14) and were used to apply details on top of background layers already painted with a different pigment; conversely, in fragments CA16 and CA20, lead-containing pigments were extensively used. Plattnerite was detected in the paint stratigraphy (almost in every lead-rich region) in all the fragments chosen for this investigation, except for fragment CA18; here, the presence of darkened vermilion was observed. Lead-containing compounds, such as scrutinyite (α-PbO_2_) and laurionite (Pb(OH)Cl), were often detected together with plattnerite (Figure 6, Figure 7, Figure 8 and Figure 9, Figures S5, S6 and S8). Considering the aesthetic and logical connection between the figures and their colors, it is plausible to assume that most of the darkened patches in the murals of the Upper Basilica originally were painted with lead white and are due to the oxidation of this latter pigment. The original white color turned brown-black as a result of the formation of plattnerite and/or scrutinyite. Remnants of hydrocerussite—the main component of lead white—were only encountered in one of the analyzed fragments (CA14); however, in this case, hydrocerussite was present in very small amounts and only in localized particles (shown in Figure S5c,f). While no red lead (Pb_3_O_4_) was observed, the lead sulfates anglesite (PbSO_4_), visible in Figure S8d, and palmierite (K_2_Pb(SO_4_)_2_), visible in Figure S5e, were identified in fragments CA14 and CA16, respectively.

*Chlorine.* As already observed by M. Vagnini et al. [4], an important Cl-K_α_ XRF signal was present in the majority of the XRF data collected from most Assisi samples; Cl is mostly correlated with the distribution of pigments in the paint layers, while lower signal levels were encountered in the substrate.

*Chromium.* Via XRF, chromium was occasionally detected: in fragment CA08, it was correlated with a blue hue and its distribution closely followed that of copper. In sample CA14, chromium was also detected; here, crocoite (PbCrO_4_) was identified (distribution and 1D XRPD pattern shown in Figure 6 and Figure S5d) both by macroscopic XRPD and in cross-sections collected from this fragment.

### 3.2. Cl-Induced Degradation of Vermilion: Corderoite and Calomel

In fragment CA18 (Figure 4), several different compounds containing mercury were identified on the paint surface and in the strata below the surface. This painted mural fragment is characterized by an abundance of vermilion and by the presence of degradation products such as corderoite and calomel, caused by the progressing interaction of the original pigment with chlorine. This aging process is described in the literature and leads to grey and brown blackened patches on the original red paint.

Vermilion derives its color from the semi-conductor pigment HgS; this corresponding mineral cinnabar was the first source of the pigment before a synthetic process was developed to produce it (8th–9th century) [17]. Cinnabar is one of the oldest known pigments ever used, and it it often found employed in burial sites all over the world [18]; its first identification in mural paintings dates back to c. 7000–8000 BC in Çatalhöyük (Turkey) [19].

In this manuscript, we referred to vermilion as the name of the applied pigment, while we have reserved the term cinnabar to describe the mineral reference(s).

While the only mercury sulfide chloride compound identified by MA-XRPD in fragment CA18 was corderoite (α-Hg_3_S_2_Cl_2_, Figure 4), the data collected from the corresponding paint stratigraphy displayed in Figure 5 (and in Figure S4c) also show the presence of lavrientivite (β-Hg_3_S_2_Cl_2_) and kenhsuite (γ-Hg_3_S_2_Cl_2_). The identification of these compounds is in strong agreement with the observations of Radepont et al. Their research investigated this process in depth, which requires both the presence of reactive chlorine species and light, leading to the degradation of the original red mercury sulfide [20]. Following their investigation of the Hg-S-Cl-H_2_O system, the presence of corderoite, lavrientivite, kenhsuite, calomel, and sulfates could be interpreted as the result of the interaction between α-HgS and ClO_(g)_, as described in the following sequence of reactions:

α-HgS ⇌ Hg_3_S_2_Cl_2_ ⇌ Hg_2_Cl_2_.

Similarly, in a more recent publication, calomel and laurionite were identified in a painting by Hieronymus Bosch called “The Saint Wilgefortis Triptych” (Galleria dell’Accademia, Venice); the conversion of original lead white and vermilion into laurionite and calomel, respectively, was monitored by employing a combination of FTIR and ToF-SIMS [21].

Part of the gypsum in fragment CA18 may have been originally present in the lime plaster and/or may have been partly formed in situ as a degradation product. In a number of cross-sectioned samples, such as the one shown in Figure 5, the µ-XRPD data show the presence of gypsum localized in a separate layer on top of the painted surface and/or within it; in these cases, a degradation phenomenon is more likely to explain its presence (see also Figure S4d). Specifically in sample CA18, the distribution of gypsum containing S in sulfate form (S^+VI^) in the upper layers suggests that it either was formed by the oxidation of the originally present sulfide ions (S^−II^) or by the deposition of S-containing gases from the ambient atmosphere [22] but not by the upward migration of sulfate ions from lower strata. In the first case, the degradation of vermilion may have given rise to the precipitation of gypsum after the reaction of SO_4_^2−^ anions with calcium cations available from the preparation layer, as highlighted in Figure 4 and Figure 5.

It is mentioned in the literature, hypochlorous acid/hypochlorite can oxidize sulfide ions to elemental sulfur; the maximum oxidation rate is observed when the pH is close to 7.5 [23]. This value is lower than the pH of a saturated solution of calcium carbonate (pH = 9) [24], where the oxidation reaction should stop. However, it may be logical to assume that in specific areas, pH levels significantly lower than 9 can be achieved due to the presence of deposited pollutants (such as HCl, SO_2_, NO_x_, and CH_3_COOH) or because of the composition of the paint.

**Figure 10:**
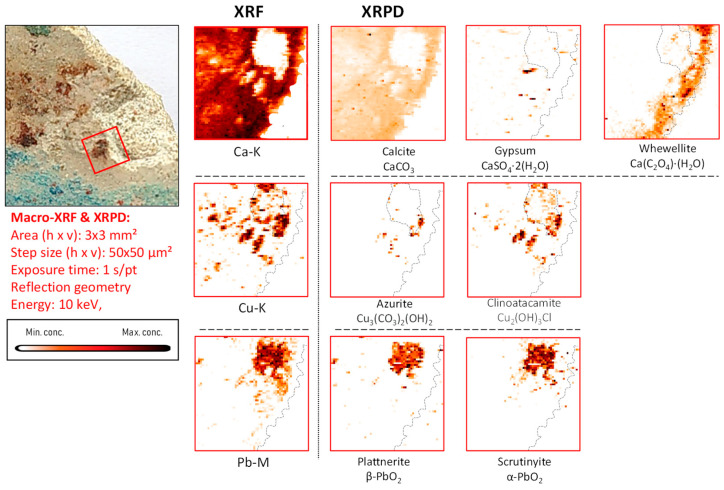
Light-blue-brown-colored vault fragment CA08, its corresponding MA-XRF maps of selected elements, and MA-XRPD maps of selected primary and secondary products; data obtained at the PUMA beamline.

**Figure 11:**
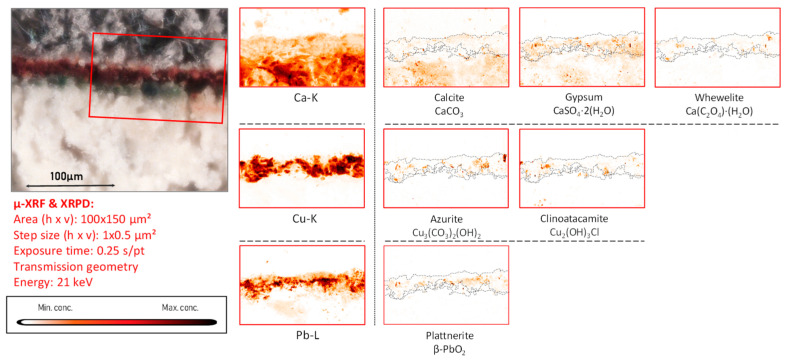
Cross-sectional micrograph of a paint sample collected from mural painting fragment CA08, its corresponding μ-XRF maps of selected elements, and μ-XRPD maps of selected primary and secondary products; data obtained at Beamline P06.

A characteristic form of substrate degradation was evidenced in fragment CA18 by the detection of diffuse distributions of calcium oxalates, shown in Figure 4 and Figure 5; these are also present in all other fragments (see Figure 6, Figure 7, Figure 8, Figure 9, Figure 10, Figure 11 and Figures S4–S9), mainly as the mono-hydrated whewellite (CaC_2_O_4_·H_2_O) or as the bi-hydrated form weddellite (CaC_2_O_4_·2H_2_O). The data derived from cross-sections show the formation of calcium oxalates localized on top of or immediately below the painted surface, where the pigment is in contact with the calcium-containing plaster, and less in paint-loss areas where calcite from the ground is now exposed. Their source has been identified in the degradation of the binder medium that reacted with the most-available chemical element in the surroundings. This agrees with the experimental work conducted by Sciutto et al. in which the oxidation of the fatty content of an egg-tempera binding medium caused the formation of calcium oxalates within and under the paint layer of wall paint samples [25].

### 3.3. Cl-Induced Degradation of Lead White: Laurionite and Plattnerite

In the mural painting fragments CA04 and CA14, laurionite (lead hydroxychloride, Pb(OH)Cl) was identified along with plattnerite (lead dioxide, β-PbO_2_).

The distribution maps obtained from sample CA04 show the extensive degradation of the original pigments caused by chlorine compounds. In this fragment, laurionite is observed to be the most-abundant compound detected in lead-rich areas. Although it is less well known in the heritage literature than the copper-hydroxychlorides atacamite and clinoatacamite, traces of its usage as a white pigment date back to the Bronze Age. The first documentation of laurionite production for artistic purposes was encountered in Akrotiri, Greece (c. 3000–1600 B.C.), where raw materials and tools for the preparation of lead-containing pigments were discovered [26]. In an experimental reconstruction aimed at investigating the manufacture of (white) pigments based on an ancient Persian text of the second half of the 12th century [27], laurionite was found to be the main compound formed. Hitherto, the intentional application of this compound in panel paintings was described in Japanese masterpieces from the 12th to the 15th century [28]; in the context of Asian mural paintings, the intentional use of laurionite as a white pigment in the Tiantishan grottoes, northwest China (397–439 B.C.), was also established [29]. However, in Europe, there is almost no tradition of using this compound. Only quite recently and for a very short period, it was exploited as a white pigment: a basic lead chloride (likely Pb(OH)Cl) was synthesized in England in the 19th century, called Pattinson’s white [1]. Considering the age of the mural paintings in the Assisi Basilica, we can only interpret the presence of laurionite here as the result of a degradation process of an original Pb-containing pigment. This agrees with the identification of laurionite previously described as a by-product of lead white in the aforementioned painting by Bosch, *the “Saint Wilgefortis Triptych”* [21].

Similarly, Hradil et al. reported the formation of laurionite in mural paintings of the 13th-century church of Saint Gallus (Northern Bohemia, Czech Republic), considering it to be a degradation product of red lead [30]. This chemical conversion is caused by a high local concentration of chlorine related to the presence of halite (NaCl) in the mortars. A similar phenomenon has been described and documented by Uchida et al. at the archaeological site of Angkor in Cambodia (1113–1150) [31].

Lead white features good covering and oil-drying properties. It is a mixture of basic lead carbonate (hydrocerussite, 2PbCO_3_·Pb(OH)_2_) and neutral lead carbonate (cerussite PbCO_3_) in different proportions and it was the most-appreciated and -common white pigment employed from ancient times until fairly recently (in the 19th century, lead white was banned because of its harmful effects) [32].

Earlier investigations carried out after the earthquake in 1997 revealed the presence of minium (Pb_3_O_4_) in fragments from the Assisi Basilica [2,3]. This compound is more frequently known as *red lead* and corresponds to the artificial equivalent of naturally occurring crystals of minium [33]; this lead (II,IV) oxide is considered to be one of the most-primitive synthetically produced pigments. The process of the solvolytic disproportionation of minium in an acidic environment has been investigated and can be described by the following reaction [34]:

Pb_3_O_4_ + 4H_3_O^+^ → PbO_2_ + 2Pb^2+^ + 6H_2_O.

However, in the present investigation, we did not encounter any Pb_3_O_4_ but instead other secondary Pb compounds such as plattnerite and anglesite (PbSO_4_), which can be derived from Pb_3_O_4_ [34]. In several studies [35,36,37], plattnerite has been identified in mural paintings, formed by the degradation of the lead-containing pigments that were originally present.

In the cultural heritage literature, it is often assumed that the oxidation process of lead white is triggered when the pigment, following the *buon fresco* technique, is exposed to the strong alkalinity of slaked lime (Ca(OH)_2_, pH > 10), causing a reduction in the redox potential, and/or when, after application, lead white becomes exposed to oxidizing agents such as sodium hypochlorite or peroxide compounds [38].

In fragment CA14, a strong co-localization of plattnerite and laurionite both at the surface and in the corresponding cross-section (Figure 6 and Figure 7) can be observed. However, the main pigment used here is iron-based, i.e., a mixture of hematite and goethite (the main mineral components of red and yellow ochre, respectively) [39], as revealed by XRPD measurements.

The different usage of these iron-containing compounds can be observed in Figure 3 (fragment CA14), in which orange and red areas are visible. Similarly to fragment CA18, in which calomel was identified, also here a secondary product containing chlorine was detected: laurionite (Pb(OH)Cl) showing the same lateral distribution as plattnerite. Laboratory experiments (not discussed in detail here) showed that the oxidation of lead white triggered by active chlorine species fosters the formation of black Pb(IV) species and lead-chlorinated compounds such as cotunnite (PbCl_2_), following the reaction below:

2PbCO_3_·Pb(OH)_2_ + 2HOCl ⇌ 2PbO_2_ + PbCl_2_ + 2H_2_CO_3_.

The relatively high solubility of cotunnite (K_sp_ ≈ 1.70 × 10^−5^) [40] and its low stability at pH levels higher than 6 causes it to be gradually converted into laurionite in a second transformation step.

Palmierite (K_2_Pb(SO_4_)_2_), (1D XRD pattern shown in Figure S5e) is a lead potassium sulfate identified in this fragment that also is considered to be a secondary product, as shown in Figure 6.

As already discussed above, calcite is clearly present in the plaster substrate, showing stronger signals in the areas with paint losses (Figure 6). However, it is additionally detected in the area still covered with a goethite-containing layer; another Ca compound, whewellite, can be observed here, which is spatially more strongly correlated with the painted areas, suggesting that its formation resulted from the oxidative degradation processes occurring in the paint layer. This is very clear in Figure 7, where the pigmented area is surrounded by a rim of calcium oxalate. In both Figure 6 and Figure 7, crocoite can be observed. While this compound is frequently encountered in modern paintings [41], in historical painting it has been identified as being associated with an intentionally applied yellow pigment employed in Northern Bohemia, perhaps as a contamination of the ochre-containing layer [30]. Considering the limited amount of crocoite identified in the Assisi fragments in general and its localization within the depth of the paint stratigraphy, the crocoite is not considered to have been applied during modern restoration.

### 3.4. Cl-Induced Degradation of Cu-Pigments: (Clino)atacamite, Cumengeite and Mixite

In mural painting fragments CA04, CA08, and CA16, where lead-containing pigments were applied by themselves or mixed with Cu-based pigments, another degradation phenomenon involving chlorine was observed to have taken place (Figure 8 and Figure S8e). This is shown in Figure 10 and Figure S6a,c, where two (Cu, Cl)-containing alteration compounds are formed in fragment CA04: atacamite and clinoatacamite. These compounds are known to have been intentionally employed as pigments in mural paintings and polychrome sculptures in Egypt [42], China [43], South America [44], and more rarely in Europe [45]. Nevertheless, the original presence of azurite, as described in Table 1, strongly suggests that these copper-chlorinated compounds are degradation products of the original pigment; this discoloration phenomenon has been widely described elsewhere [46,47]. Additionally, it is known that natural azurite ores can contain zinc and arsenic (usually in the form of zinc arsenates), bismuth, and barium [48]; the presence of the latter elements was confirmed by XRF data in fragments CA04 and CA08.

Sample CA04 was of particular interest because the lead- and the copper-containing areas show a significant alteration of the original pigment and because it had already been analyzed by means of vibrational analytical techniques, highlighting the oxidation of lead white to lead dioxide [4]. In the green areas, mostly clinoatacamite (Cu_2_(OH)_3_Cl) was encountered together with azurite, illustrating once more the degrading influence of chlorine compounds on the original applied pigments (shown in Figure S6a,b).

In the non-blackened parts of the surface of this fragment, by means of MA-XRPD analysis, the mineral cumengeite (21PbCl_2_·20Cu(OH)_2_·6H_2_O), a mixed (Pb,Cu) basic chloride [49] salt rarely found in paintings, was localized (see Figure 8). This compound was discovered in 1893; it is composed of 20 copper centers forming a flattened cuboctahedron and has been subject to in-depth investigations [50]. Cumengeite has been identified in historical bronzes containing lead as a degradation patina [51] and in a historical Roman coin at the grain boundaries in lead-rich zones together with laurionite and mendipite (Pb_3_Cl_2_O_2_) [52]. Hradil et al. similarly identified cumengeite in a wall painting as an original (and thus intentionally applied) blue pigment; since these authors noted a lack of lead-containing compounds in the vicinity of the area where cumengeite was found, they excluded the possibility of its in-situ formation by paint degradation processes [48].

In nature, the formation of cumengeite occurs in complex lead–copper mineral assemblages in oxidized zones [50]. Due to its rarity in the natural environment and the difficulties in collecting significant amounts of the mineral to apply it for artistic purposes, a historical recipe for the preparation of this blue pigment has been proposed as a way to explain its identification in works of art [48].

Nevertheless, from the XRF and the XRPD maps shown in Figure 8, an abundant combined presence of lead- and copper-chlorinated compounds can be observed, making the hypothesis of the in-situ formation of cumengeite plausible. Recent investigations involving laboratory synthesis experiments performed by the authors have allowed us to shed more light on this: we have effectively observed the in-vitro formation of cumengeite when Pb and Cu carbonates are exposed to hypochlorous acid in alkaline conditions (unpublished results).

In mural painting fragment CA04, a second exotic Cu compound was encountered below the painted surface (Figure S6c,e): mixite (BiCu_6_(AsO_4_)_3_OH_6_·3H_2_O). This rare compound naturally occurs as a secondary mineral in the oxidized zones of copper deposits [53]. It was found to be present by Berrie et al. in “Madonna with Child”, as painted by Giotto [54]. Being a mixed (Cu,Bi) arsenate, it belongs to the same family of (fairly) insoluble arsenates that have recently been encountered in works of art from later periods and that result from the oxidation of arsenic sulfides [55,56,57]. As shown in Figure S6c, next to the secondary product mixite, and the Cl-containing degradation products laurionite, cumengeite, and clinoatacamite were also detected in this paint cross-section.

### 3.5. Plattnerite β-PbO_2_ and Scrutinyite α-PbO_2_

In three of the investigated samples (CA08, CA16, CA20), in addition to plattnerite (β-PbO_2_), another allotropic form of PbO_2_ is encountered: scrutinyite (α-PbO_2_). Lead dioxide is polymorphic, and the properties of three phases are well documented in the literature: an orthorhombic phase (α-PbO_2_, with a columbite structure), a tetragonal phase (β-PbO_2_, with a rutile structure), and a high-pressure γ-modification. Both α-PbO_2_ (space group Pbcn) and β-PbO_2_ (space group P42/mnm) are stable at room temperature and pressure, with the β-phase being the most stable in neutral/acid conditions, while α-PbO_2_ is favored in alkaline environments [58], as described by the following equations:

Pb^2+^_(aq)_ + Cl_2(g)_ + 2H_2_O ⇌ β-PbO_2(s)_ + 2Cl^−^_(aq)_ + 4H^+^_(aq)_

Pb^2+^_(aq)_ + Cl_2(g)_ + 4OH^−^_(aq)_ ⇌ α-PbO_2(s)_ + 2H_2_O + 2Cl^−^_(aq)_

As can be gleaned from the standard reduction potentials of the half-reactions mentioned below, there is only a slight thermodynamic difference in stability between scrutinyte and plattnerite in standard conditions [59]:

β-PbO_2(s)_ + 4H^+^_(aq)_ + 2e^−^ ⇌ Pb^2+^_(aq)_ + H_2_O, E° = +1.460 V vs. Standard Hydrogen Electrode;

α-PbO_2(s)_ + 4H^+^_(aq)_ + 2e^−^ ⇌ Pb^2+^_(aq)_ + H_2_O, E° = +1.468 V vs. Standard Hydrogen Electrode.

Thus, it is unsurprising that the dissolution reactions of both phases are also characterized by equilibrium solubility products constants of similar magnitude, only very slightly favoring the precipitation of β-PbO_2_ [60]:

β-PbO_2(s)_ + 4H^+^ ⇌ Pb^4+^_(aq)_ + 2H_2_O  log K_sp_ = −8.91

α-PbO_2(s)_ + 4H^+^ ⇌ Pb^4+^_(aq)_ + 2H_2_O  log K_sp_ = −8.26

Hence, tetragonal or β-PbO_2_ (plattnerite) is thought to be in the most stable form at room temperature and pressure [61], while in many practical situations, the co-precipitation of both forms may occur. Empirically, β-PbO_2_ has often been detected during investigations of discoloration phenomena affecting both white and red lead [34,35,36,37,38], while scrutinyite has been identified in fewer instances [62,63]. The formation of scrutinyite has been linked to the lead white subtype it forms from. Vagnini et al. and Avranovich et al. have previously observed that cerussite-rich types of lead white, when oxidized by dilute NaOCl, tend to form plattnerite, while hydrocerussite-rich lead whites favor the formation of scrutinyite [4,63,64]. In a different context, Wang et al. examined the oxidation of pure cerussite and hydrocerussite by sodium hypochlorite solutions: depending on the precursor identity, respectively scrutinyite and/or plattnerite are formed after the reaction [60].

The correspondence between the lateral distributions of scrutinyite and plattnerite in sample CA08 are evident in Figure 10, confirming that they were formed as co-precipitates and as a result of the same alteration process; in fragments CA16 and CA20, such a spatial correlation can also be observed (see Figures S8 and S9).

In general, we interpret the presence of both polymorphs of Pb(IV) oxide without traces of Pb(II) as an indicator of fairly aggressive oxidation conditions in which the redox equilibria involved are completely shifted to the right, i.e., in favor of the Pb(IV) compounds.

The presence of clinoatacamite, clearly spatially correlated to that of azurite in fragment CA08 (Figure 10 and Figure 11), again underscores the degrading influence of Cl compounds. However, apart from the two PbO_2_ species, no laurionite was encountered on the surface of this fragment. We interpret this absence as being caused by a more complete oxidation of the lead white in which the intermediate compound laurionite dissolved and the resulting Pb^2+^-ions completely converted into Pb^4+^-species.

## 4. Discussion

Many of the above-described changes can directly or indirectly be attributed to the common oxidative influence of chlorine compounds on the original pigments. In areas originally painted with vermilion, the formation of several Hg_3_S_2_Cl_2_ polymorphs, calomel, and gypsum was observed; while the first compounds are known to whiten the red hue of vermilion, the latter can be considered responsible for the darkening of these areas, because of its structure and its capability to entrap (black) carbon particles in its pores [65]. In areas of darkened lead white, compound-specific distributions obtained through XRPD mapping highlighted the presence of either plattnerite and/or scrutinyite, sometimes together with laurionite. These data suggest that laurionite, well evidenced in localized areas (see Figure S5d), may have been first produced as a result of simple ion exchange between chlorine compounds and lead white, reacting further in a second stage to either scrutinyite or plattnerite, depending on the alkalinity of the environment. The (slightly) more unstable scrutinyite could then finally be converted into plattnerite. In previous studies on model samples [4,60,65], the observed oxidation of lead white has invariably been encountered in contexts where oxidizing chlorinated compounds were present. While it is not completely clear what the source of the reactive chlorine species is, the most plausible scenario is that reactive and gaseous Cl compounds present in disinfecting/cleaning products, such as NaOCl solutions, are the main common triggering factor for the degradation of the lead white, azurite blue, and vermilion red on the Basilica ceiling and murals. In addition, reactive species of chlorine are also known to be the main active ingredients in biocides applied in wall cleaning and these products may have been used in the past (by the monks) in the Basilica. Alternative causes such as Cl-containing salts in the mortar of the church walls and Cl compounds formed by combustion processes (e.g., in the building heating system, during incense burning) appear less likely as they do not cause the formation of strongly oxidized secondary compounds such as plattnerite or scrutinyite. Given the consistency of the degradation phenomenon observed in three different pigments, it is considered crucial in the future to avoid the use of NaOCl or similar substances in the indoor environment of the church.

The discoloration of blue areas to green in the vault of the Assisi Basilica can be linked to the formation of secondary Cl-containing compounds such as atacamite and clinoatacamite. In addition, the rarer minerals cumengeite and mixite could also be identified, and these species have been similarly considered as secondary products and linked to a common aggressive degradation mechanism. It is hypothesized that this phenomenon can affect more pigments and, depending on their solubility, be shared by different chemical species, causing reiterative interaction between the reagents.

The cause for the formation of calcium oxalates commonly identified in all the painted fragments has been correlated with the application of the pigments employing a lipidic binder, such as egg tempera [4], and with an aggressive oxidative process, similar to the one just described. Oxidative stress can favor the destruction of the lipid-containing organic binder and the formation of oxalic acid, as suggested by Colombini et al. [66]. In addition, interesting similarities were highlighted between the degradation behavior of inorganic pigments applied in Northern Bohemia as identified by Hradil et al. [30,31,32,33,34,35,36,37,38,39,40,41,42,43,44,45,46,47,48] and those of the same historical period and a similar context identified within the mural painting fragments of the Assisi Upper Basilica. A common source of azurite and ochre may have been exploited by the painters in both locations, as shown, for example, by the azurite detected in the monastery of Sázava (14th century) that contains Zn- and/or Cu-arsenate impurities in the form of granules. Likewise, the presence of crocoite in the ochre-colored layers from Assisi is similar to that identified in paint fragments from the church of Saint Gallus (Bezděz Castle in Northern Bohemia) painted at the end of the 13th century.

## 5. Conclusions

In this paper, the strong discoloration processes that took place in the wall paintings of the Basilica of Saint Francis in Assisi were discussed. Thanks to the opportunity to analyze various fragments of mural painting recovered after the earthquake in 1997, it was possible to correlate and find similarities in the degradation mechanisms of all the main pigments examined.

1. In all the fragments, the presence of various secondary products such as corderoite, plattnerite, calomel, laurionite, clinoatacamite, and atacamite, almost always containing chlorine, was commonly identified.
2. The identification of the lead–copper hydroxychloride cumengeite strongly suggested a synergistic effect in the degradation of the originally applied copper- and lead-containing compounds, and it might act as a chlorine reservoir for the further degradation of the originally applied pigments when a local variation in pH occurs.
3. The degradation has not only affected the inorganic pigments but also the substrate and the impasto medium used to create the murals. The formation of gypsum and calcium oxalates such as whewellite and weddellite was equally identified in all samples, and these were considered by-products. The former is due to the calcium-containing substrate interaction with the atmosphere and/or vermilion, and the latter is due to the reactivity of the substrate with the binder.

X-ray fluorescence (XRF) and X-ray diffraction (XRPD) imaging have shown their pivotal role in their ability to localize and identify the intentional pigments applied by the artist and their degradation products. While canvas paints are prone to yellowing and protective varnish is prone to deterioration, in comparison, wall paints have shown a lower ability to protect and preserve the intentionally applied inorganic pigments. The alkaline substrate increases the vulnerability of pigments to degradation and can cause strong color variations. The noticeable color changes of the originally applied pigments such as azurite, lead white, and vermilion are related not only to the nature of the degradation process but also to the fact that their use in mural paintings was strongly discouraged in manuscripts about painting techniques. It is significant to underline that, while largely based on empirical observations, advanced technical knowledge about the stability of inorganic pigments was already available and that such insights were shared among artists of the Middle Ages, sometimes in the form of guild regulations. However, the caveats about avoiding employing certain artists’ pigments in combination with the alkaline substrate for wall paintings apparently were not always followed. In addition, it must have been very difficult for Old Masters to foresee the long-term effect of environmental factors such as oxidative chlorine compounds on their creations and adapt their pigment usage accordingly.

## Figures and Tables

**Table 1:** Chemical elements and crystalline compounds identified in all examined paint fragments and cross-sections. The major chemical elements identified are indicated in **bold**.

Plaster Fragment	Location	Description	Elements Identified (XRF)	Crystalline Compounds Identified (XRPD)
CA04	Saint Matthew	St. Matthew’s blue mantle: yellow ground layer, blue black alteration	S, **Cl**, K, **Ca**, Ti, Mn, **Fe**, Ni, **Cu**, As, Sr, Ba, **Pb**, Cr, Zn, Bi	Clinoatacamite, plattnerite, whewellite, gypsum, laurionite, cumengeite, azurite, goethite, mixite, calcite
CA08	Saint Matthew	black and blue book on the desk	S, Cl, K, **Ca**, Ti, Cr, Mn, Fe, Ni, **Cu**, Zn, As, Sr, Ba, **Pb**, Bi	Azurite, gypsum, plattnerite, calcite, whewellite, clinoatacamite, scrutinyite
CA14	Saint Matthew	yellow desk with a stripe	S, Cl, K, **Ca**, Ti, Mn, **Fe**, Sr, Sn, **Pb**, Cr, Sn	Goethite, gypsum, laurionite, plattnerite, whewellite, hematite, calcite, hydrocerussite, crocoite, palmierite,
CA16	Unknown	black alteration, likely from dome architecture	S, Cl, **Ca**, K, Ti, Cr, Mn, Fe, Cu, Sr, **Pb**	Plattnerite, scrutinyite, hematite, whewellite, gypsum, calcite, weddellite, atacamite, anglesite
CA18	Unknown	brown alteration, likely from dome architecture	**S**, **Cl**, **Ca**, K, Ti, Mn, Fe, Sr, **Hg**	Cinnabar, calomel, cordeorite, whewellite, gypsum, calcite, weddellite, lavrientivite, kenhsuite
CA20	Unknown	black-pink alteration, perhaps from architectural detail	S, Cl, **Ca**, K, Mn, Fe, Sr, Sn, Ba, **Pb**	Plattnerite, scrutinyite, hematite, weddellite, whewellite, gypsum, calcite

## Data Availability

Processed data can be found together with the supplementary information at: https://zenodo.org/record/8246220 (accessed on 10 August 2023).

## Supplementary Materials

The following supporting information can be downloaded at: https://zenodo.org/record/8246220 (accessed on 10 August 2023). Refs. [67,68,69,70] are cited in the Supplementary Materials.

## Sample Availability

Vittoria Garibaldi, formerly from the Soprintendenza per i Beni Storici Artistici e Etnoantropologici dell’Umbria has allowed access to the fragments of mural painting.
